# The Language of Compassion: Hospital Chaplains’ Compassion Capacity Reduces Patient Depression via Other-Oriented, Inclusive Language

**DOI:** 10.1007/s12671-022-01907-6

**Published:** 2022-05-30

**Authors:** Jennifer S. Mascaro, Patricia K. Palmer, Madison Willson, Marcia J. Ash, Marianne P. Florian, Meha Srivastava, Anuja Sharma, Bria Jarrell, Elizabeth Reisinger Walker, Deanna M. Kaplan, Roman Palitsky, Steven P. Cole, George H. Grant, Charles L. Raison

**Affiliations:** 1 Department of Family and Preventive Medicine, Emory University School of Medicine, 1841 Clifton Road NE, Suite 507, Atlanta, GA 30329, USA; 2 Department of Spiritual Health, Emory University Woodruff Health Sciences Center, Emory Healthcare, Atlanta, GA, USA; 3 Department of Behavioral, Social, and Health Education Sciences, Rollins School of Public Health, Emory University, Atlanta, GA, USA; 4 Graduate Division of Religion, Emory University, Atlanta, GA, USA; 5 Department of Behavioral and Social Sciences, Brown University, Providence, RI, USA; 6 Research Design Associates, Inc, Yorktown Heights, NY, USA

**Keywords:** Compassion, Spiritual health, Chaplain, LIWC, Skillful means, Depression

## Abstract

**Objectives:**

Although hospital chaplains play a critical role in delivering emotional and spiritual care to a broad range of both religious and non-religious patients, there is remarkably little research on the best practices or “active ingredients” of chaplain spiritual consults. Here, we examined how chaplains’ compassion capacity was associated with their linguistic behavior with hospitalized inpatients, and how their language in turn related to patient outcomes.

**Methods:**

Hospital chaplains (*n* = 16) completed self-report measures that together were operationalized as self-reported “compassion capacity.” Next, chaplains conducted consultations with inpatients (*n* = 101) in five hospitals. Consultations were audio-recorded, transcribed, and analyzed using Linguistic Inquiry Word Count (LIWC). We used exploratory structural equation modeling to identify associations between chaplain-reported compassion capacity, chaplain linguistic behavior, and patient depression after the consultation.

**Results:**

We found that compassion capacity was significantly associated with chaplains’ LIWC clout scores, a variable that reflects a confident leadership, inclusive, and other-oriented linguistic style. Clout scores, in turn, were negatively associated with patient depression levels controlling for pre-consult distress, indicating that patients seen by chaplains displaying high levels of clout had lower levels of depression after the consultation. Compassion capacity exerted a statistically significant indirect effect on patient depression via increased clout language.

**Conclusions:**

These findings inform our understanding of the linguistic patterns underlying compassionate and effective chaplain-patient consultations and contribute to a deeper understanding of the skillful means by which compassion may be manifest to reduce suffering and enhance well-being in individuals at their most vulnerable.

Compassion is central to the practice of medicine (Paterson, 2011) and evidence is mounting that compassionate care is associated with improved patient outcomes (Derksen et al., 2013; Fogarty et al., 1999; Kelley et al., 2014; Lelorain et al., 2012; Rakel et al., 2009; Trzeciak & Mazzarelli, 2019). Hospital chaplains play a vital role in delivering compassionate emotional and spiritual care to a broad range of both religious and non-religious patients (Handzo et al., 2008) for a wide variety of stressors (Vanderwerker et al., 2008). As the responsibilities of social workers grow well beyond psychosocial support to include discharge procedures, financial aid, insurance, and other social needs, chaplains’ purview over attending to the suffering of patients and their families may be increasingly relevant. Moreover, chaplains encounter not only the physical and psychological distress of patients, but also the doubts, fears, and existential pain that come when illness strains patients’ beliefs. In addition to caring for patients and family members, chaplains also bear witness and provide care to hospital staff who themselves may experience secondary stress or moral injury from their exposure to patients’ suffering, or who are struggling with other stressors in their life or work (Fitchett, 2017).

Extensive research indicates that chaplain consults can positively impact patient outcomes (Flannelly et al., 2012; Jankowski et al., 2011), well-being (Kevern & Hill, 2015), and satisfaction (Marin et al., 2015; Pesut et al., 2016). Although this accumulating body of scholarly work highlights the value of spiritual care consults, remarkably little research has been conducted to identify the “active ingredients” of chaplaincy spiritual care, with the result that the field lacks standardization and best-practice guidelines for informing chaplain spiritual care consulting (Handzo et al., 2014; Jankowski et al., 2011; Massey et al., 2015; Montonye & Calderone, 2010). The need for rigorous research in this area is highlighted by the demands on modern-day chaplains to address broader emotional and social dimensions of care (Pesut et al., 2016).

Chaplains learn to deliver inclusive and skillful spiritual care in Clinical Pastoral Education (CPE) programs, which bring theological trainees of all faiths into supervised encounters with persons in the healthcare system experiencing a crisis. In addition to formal instruction, CPE trainees receive informal feedback from peers and educators, such that trainees develop a new awareness of themselves, interpersonal and inter-professional skills, and appreciation of the needs of those for whom they care. Like clinical psychologists and social workers, chaplains are trained to recognize and respond to emotional distress and to provide counseling. Chaplains are also uniquely and explicitly trained to recognize and respond to spiritual distress and to assist patients in exploring religious or worldview-based values and resources that can be brought to bear in coping with illness, making medical decisions, and reducing suffering. CPE trains chaplains to recognize religious, spiritual, and emotional needs, identify and apply resources, and integrate the spiritual domain into whole-person, patient-centered care for those of any or no religion. This training is critical, as spiritual care requires chaplains to maintain a state of vulnerability in the face of others’ suffering, and for this reason, their training in emotional intelligence and compassion is thought to be central to mitigating burnout and compassion fatigue (Galek et al., 2011). In fact, hospital chaplains appear to be particularly resilient in their ability to maintain compassion and low levels of burnout even while providing care to high-acuity patients experiencing profound distress (Ash et al., 2020; Oliver et al., 2018; Taylor et al., 2006; Yan & Beder, 2013), although there is some evidence that younger chaplains are at higher risk for burnout (Yan & Beder, 2013). For these reasons, CPE is designed in large part to cultivate the psychosocial skills and attributes that hospital chaplains, as spiritual health clinicians, need to deliver skillful compassion.

While compassion is considered foundational to the effective clinical interaction (Tehranineshat et al., 2019; Trzeciak & Mazzarelli, 2019), far less is known about the behaviors that convey compassion in a clinical environment. What are the factors that cultivate a therapeutic alliance, create a healing context, and help a patient feel figuratively held in care? Early influential research found that patients commonly present clues about their emotional and social concerns, but physicians frequently miss the opportunity to respond (Levinson et al., 2000; Suchman et al., 1997). Interestingly, some of these studies found that longer patient visits were more—not less—likely to contain missed opportunities, indicating that time burden cannot be the only explanation. However, other studies have found the opposite relationship, suggesting that time demands may sometimes interfere with compassionate and relational communication (for example, (Marvel, 1993). A more recent set of studies has produced an influential model for compassion in healthcare using mixed-methods approaches to examine clinical compassion from diverse patients’ perspectives (Sinclair et al., 2016, 2017; Singh et al., 2018). In general, these studies have characterized clinical compassion as a multi-faceted construct that includes the virtues, qualities, and beliefs of the care provider, the inter-relational understanding between care provider and patient, and the active delivery of emotional and physical support in response to the patient’s suffering. Extending this research to examine the provider characteristics associated with compassionate behavior, there is some evidence that emotional intelligence and secure attachment style are associated with more optimal relational and compassionate person-centered care (Cherry et al., 2013). However, very little research has been conducted to understand the specific aspects of behavior that convey compassion and help confer a psychosocial benefit to patients during a clinical encounter.

In the clinical science field, interpersonal elements such as provider warmth, genuineness, empathy, expertise, and respect have been identified as “common factors” that strongly predict patient outcome regardless of the content or theoretical orientation of psychosocial interventions (Cuijpers et al., 2019). Some extant research provides insights into how these non-specific elements may be conveyed. If a chaplain’s compassion leads to improved patient outcomes, one way that it may do so is via the language the chaplain uses in the consultation (Singh et al., 2018). For example, there is a growing body of research that links pronoun use from family, caregivers, and providers with positive health outcomes among patients (Falkenstein et al., 2016; Rohrbaugh et al., 2008, 2012). The theoretical interpretation of this set of findings is that pronouns often act as “stealth words” that can reveal to whom one is paying attention, regardless of the topic of conversation (Pennebaker, 2011). In the clinical context, the use of more inclusive and other-oriented pronouns may function to convey empathy and compassion, which may then confer a benefit to the patient. Another linguistic behavior that may be associated with chaplain compassion and serve to create a healing context is affective language. For example, a previous study found that patients report liking their physician more if the provider used fewer negative emotion words (Falkenstein et al., 2016). Together, this research indicates that subtleties of spoken language may be a fundamental—though by no means only—way that non-specific therapeutic factors such as empathy, compassion, and interpersonal attunement are conveyed and cultivated within the dyadic clinical relationship.

The aim of this study was to examine how chaplain compassion manifests in bedside behavior with patients, and how bedside behavior (operationalized via linguistic markers) confers the benefits that patients experience. Specifically, we examined the relationship between chaplain-reported compassion capacity, chaplain language during spiritual health consultations, and patient depression after the consultation. We hypothesized that chaplains’ self-reported burnout, compassion satisfaction, and medical empathy would comprise a “compassion capacity” latent variable. We further hypothesized that compassion capacity would be associated with chaplain language during their consultations with hospitalized patients and with patient depression after the consultations, controlling for pre-consultation distress levels.

## Methods

### Participants

Chaplain residents were recruited from full-time, year-long trainees enrolled in a Clinical Pastoral Education (CPE) program accredited by ACPE: The Standard for Spiritual Care and Education. There were no exclusion criteria for the chaplain residents. The social and demographic characteristics of chaplain residents (*N* = 16) are presented in Table 1. Chaplains conducted between 2 and 11 consultations with patients (mean [*M*]: 6.25, standard deviation [*SD*]: 2.28), with 6 as the modal number of consultations.

Chaplain residents in this CPE program are assigned to one of five hospital locations for most of their instructional and clinical activities. Within these five hospital locations, chaplains and chaplain residents provide consultations to patients of any or no faith, and they attempt to consult with all inpatients when first admitted. In addition, chaplain consultations occur at the request of the patient or family, and at the request of clinical staff when they are concerned about a patient’s well-being. Chaplains also respond to cardiac arrest codes and deaths, and they assist patients in the completion of advance directives and in end-of-life planning. Chaplains in this healthcare system averaged 72,811 patient consultations per year from 2017 to 2020, and they conducted 77,683 consultations with patients in 2019.

Patient participants were recruited between August 2018 and March 2019 from five acute-care hospitals in a major metropolitan area in the southeastern USA. Patients were eligible for inclusion in the study if they were at least 18 years of age, English speaking, and receiving care on an inpatient unit (unit types and patient demographics can be found in Table 2). Patients were excluded if they were determined by the researcher to be cognitively impaired (e.g., unable to answer basic questions), on a ventilator, or were in a room requiring enteric precautions or airborne precautions (use of an N-95 mask requiring fit testing) to enter. The clinical, social, and demographic characteristics of patients (*N* = 101) are presented in Table 2. Patients were 56% female and 44% male, with a mean age of 59.3 (*SD*: 17.2; range: 20.6–91.7).

### Procedures

As part of a larger study to evaluate a compassion-centered spiritual health program, we recruited chaplain residents (*n* = 16) and the hospitalized patients they consulted (*n* = 101). The study was approved by the Emory University Institutional Review Board and all participants (chaplains and patients) gave written informed consent. First, chaplain residents completed self-report measures to assess their compassion capacity. Next, in separate sessions spanning 6 months, researchers shadowed chaplain residents as they conducted consultations with hospitalized inpatients. Prior to the chaplain entering the room for the consultation, a member of the research team conducted informed consent with the patient. If the patient consented to participate in the study, the researcher administered a single-item distress measure (described below), and then left the room while the chaplain resident interacted with the patient. The chaplain resident was given an audio recorder, placed in their pocket, to record the consultation. After the chaplain resident completed the consultation and left the patient’s room, a member of the research team administered the post-consult patient-reported outcome measures.

### Measures

#### Chaplain Self-Report Measures

We adopted an approach that treats clinical compassion as not only multi-faceted, but also as composed of multiple active ingredients (Halifax, 2012; Strauss et al., 2016). Therefore, the application of a single measure was eschewed in favor of a latent variable approach, which tapped into vital but distinct dimensions that all contribute to clinical compassion, influentially defined as “a virtuous response that seeks to address the suffering and needs of a person through relational understanding and action” (Sinclair et al., 2016). First, we were interested in chaplains’ compassion satisfaction, or the pleasure chaplains derive from their work helping others (Stamm, 2005). Second, we included a construct in many ways antithetical to compassion satisfaction, namely, burnout (the feeling of hopelessness and cynicism in helping others effectively) (Stamm, 2005). Third, we included a measure of medical empathy that taps into chaplains’ beliefs about the importance of empathy in a clinical setting (Hojat & LaNoue, 2014; Hojat et al., 2009, 2015). Empathy is both conceptually and phenotypically distinct from compassion (Goetz et al., 2010; Sinclair et al., 2017; Singer & Klimecki, 2014), defined as an emotional response to another’s suffering that acknowledges and attempts to understand what the other is feeling. However, empathy is often thought to be a fundamental component of and precursor to compassion (Patel et al., 2019; Singer & Klimecki, 2014), and measures of medical empathy are highly predictive of behavior in previous studies (Berg et al., 2011; Hojat et al., 2011, 2020). Together, these measures tap into the pleasure chaplains derive from compassionate clinical care, the extent to which they feel hopeless and cynical about their ability to care for patients, and their beliefs about the importance of empathy in a clinical context.

We measured chaplains’ compassion capacity as a latent variable using the following psychometric scales. First, we administered the Professional Quality of Life version 5 (ProQOL 5) (Stamm, 2005). The ProQOL is a 30-item inventory that measures the negative and positive effects of helping others who experience suffering. The ProQOL has subscales for compassion satisfaction, burnout, and compassion fatigue (10 items in each subscale) and asks participants to indicate on a Likert scale (1 = “Never” to 5 = “Very often”) how frequently they experienced various negative and positive emotions, thoughts, and beliefs over the last 30 days as a healthcare professional. Here, we focused on two subscales: (1) compassion satisfaction, or the pleasure one derives from professional activities related to helping others (e.g., “I feel invigorated after working with those I help”); and (2) burnout, or the feeling of hopelessness and difficulty in helping others effectively (e.g., “I feel “bogged down” by the system”). While the ProQOL also includes a subscale measuring compassion fatigue, this subscale includes and is highly correlated with the burnout subscale. To avoid duplicative variables, we chose to only include the burnout subscale. Cronbach’s *α* indicated good internal reliability for each subscale: compassion satisfaction *α* = 0.85, burnout *α* = 0.88.

In addition, we administered the Jefferson Scale of Empathy (Hojat et al., 2009, 2015), a 20-item scale designed to measure empathy in practicing healthcare professionals and healthcare professional students. Here, we used the Health Professions Student version (HPS version) of the Jefferson Scale. Participants respond using a Likert scale (1 = “strongly disagree” to 7 = “strongly agree”) to indicate their level of agreement with statements about the importance of empathy in a clinical setting (e.g., “I believe that empathy is an important factor in patients’ treatment”). Cronbach’s *α* indicated poor internal reliability, *α* = 0.34. Although it is unclear why the instrument performed poorly in this population, it is likely due to the relatively small sample of chaplain residents (Bujang et al., 2018). We elected to include this measure as it has demonstrated good reliability in previous studies (Hojat & LaNoue, 2014).

#### Patient Self-Report Measures

Prior to the chaplain consultation, we administered the National Comprehensive Cancer Network Distress Thermometer (Jacobsen et al., 2005), a single-item distress thermometer that compares favorably with longer measures used to screen for distress. The distress thermometer asks patients to indicate how much distress they have been experiencing in the past week, including that day using a scale from 0 (“No distress”) to 10 (“Extreme distress”).

Upon completion of the chaplain consultation, we administered the Hospital Anxiety and Depression Scale (HADS) (Snaith, 2003; Stern, 2014; Zigmond & Snaith, 1983), a 14-item questionnaire that is widely used for detecting anxiety and depressive disorders in a hospital setting. Participants use a 4-point (0 = “Not at all”; 3 = “Nearly all the time”) response category to rate how they have been feeling in the past week, such that possible scores range from 0 to 21 for depression (e.g., “I feel as if I am slowed down”). Cronbach’s *α* indicated good internal reliability, *α* = 0.81.

#### Audio Recordings

For each chaplain-patient interaction, the audio recording was transcribed and then checked by two independent researchers, with discrepancies resolved by the research team as needed. Text preparation included the modification of transcripts into three different formats: chaplain + patient speech, chaplain-only speech, and patient-only speech. Here, we focused on chaplain-only speech (other results will be reported in a separate manuscript). Grammatical changes were performed using spell check on patient and chaplain speech to ensure a wider opportunity for LIWC 2015 to capture speech. For example, phrases such as “ain’t” were modified to “are not.”

#### Linguistic Analysis

To analyze the language recorded during the chaplain consultations, we used the LIWC 2015 software (Pennebaker et al., 2015), a widely used and extensively validated word count–based text analysis tool in the social sciences. LIWC counts words by matching them from the text to an internal dictionary, and then calculating the percentage of total words that match a set of pre-determined grammatical and semantic domains, such as affect words (e.g., sadness), social words (e.g., family), core drives and needs (e.g., achievement), and biological processes (e.g., body). The variables based on these analyses are expressed as the percentages of all words spoken by the chaplain in that consultation.

In addition to the relative frequency of words from each domain, LIWC generates four summary variables for each text file: analytical thinking, clout, authenticity, and emotional tone. Scoring for each of the summary variables is based on previously published findings in which several linguistic categories are aggregated and converted to a standardized percentile score that can range from 0 to 100 (Pennebaker et al., 2015). Analytic thinking is thought to reflect a greater degree of formal or logical thinking styles on the part of the speaker, and it is calculated based on the formula: [articles + prepositions—pronouns—auxiliary verbs—adverb—conjunctions—negations] (Jordan et al., 2019). Clout is thought to reflect the expressive confidence of the speaker and it is calculated based on the formula: [we-words + you-words + social words—i-words—swear words—negations—differentiation words] (Jordan et al., 2019). Authenticity is a measure of the degree to which someone is personal, vulnerable, or humble in their speaking. Emotional tone is a measure of the relative ratio of positive to negative emotion words, such that the higher the number, the more positive the tone. For this study, we focused on the four summary variables to reduce problems related to multiple comparisons, to reduce the number of observable variables in our exploratory SEM, and because these variables aggregate the linguistic categories of greatest interest to our study, including pronoun use, affective language, and the use of social words.

### Data Analyses

The data were analyzed using Statistical Package for the Social Sciences software, version 26.0. We used expectation maximization (Graham, 2009) to estimate missing items (missing items never accounted for more than 5% of total data) using other items within the scale as predictor variables. Assumption of distribution normality was assessed with the Shapiro–Wilk test. With a statistically significant Shapiro–Wilk test, bootstrap simulation using 500 samples was used to assess the normality of the underlying sampling distribution of the measure to determine if the use of parametric statistical tests based on the Central Limit Theorem would be appropriate. Pearson’s product-moment correlations were conducted between all study measures.

We conducted structural equation modeling (SEM) using IBM SPSS Amos v. 28 (Arbuckle, 2014) to understand how several paths when combined could predict how chaplain compassion capacity and consultation language impact patient depression. Goodness-of-fit of the SEM models was assessed with indices widely used in the applied literature (Byrne, 2016): For models with about 75 to 200 cases, the chi-square test is generally a reasonable measure of fit (Kenny, 2020); root mean square of approximation (RMSEA) and the comparative fit index (CFI) are also helpful measures of fit. Because fit indices can be affected by various analytic attributes such as sample size and estimation method, absolute fit index cutoffs are not recommended. However, guidelines for a reasonably good fit between a SEM theoretical model and observed data have been suggested: chi-square *p* value greater than 0.05; RMSEA value less than 0.10 for a low degree of freedom models (Kenny, 2020); and CFI values 0.95 or greater (Hu & Bentler, 1999), although CFI values in the range of 0.90 and 0.95 may be indicative of an acceptable model fit (Bentler, 1990). Bootstrapping analyses with bias-corrected 95% confidence intervals were performed to estimate the indirect effect of compassion capacity on patient depression via consultation language.

Prior to testing our proposed pathways between compassion capacity, language, and patient depression, we generated standardized residuals for the patient depression measure to control for pre-consultation levels of distress. We did this using linear regression, with depression as the dependent variable and scores on the pre-consultation distress thermometer as the independent variable. Tests of significance were two-sided, and results with *p* ≤ 0.05 were considered statistically significant.

## Results

Chaplain compassion capacity was measured as a latent variable comprised of compassion satisfaction, burnout, and empathy. Scores on the compassion satisfaction subscale of the ProQOL ranged from 31 to 47 on the 10–50 scale (*M*: 40.4; *SD*: 5.52). Scores on the burnout subscale ranged from 14 to 37 out of a possible 10–50 (*M*: 21.7; *SD*: 6.38). Scores on the Jefferson scale of empathy ranged from 103 to 128 out of a possible 20–140 (*M*: 115.6; *SD*: 6.69).

Patient distress levels as measured by the distress thermometer prior to chaplain consultation ranged across the entire scale, from 0 to 10, with a mean score of 5.94 (*SD*: 3.25) and a modal score of 10. Seventy-five (74%) patients scored a 4 or higher, the cutoff most commonly used to indicate clinically significant levels of distress (Donovan et al., 2014). Patient HADS depression scores after the consultation ranged from 1 to 20 out of a possible 0 to 21, with a mean score of 5.20 (SD: 4.25). Seventy-seven (77%) patients scored in the 0–7 range indicating no depression after the consultation. Seven patients (7%) scored in the 8–10 range indicating mild depression, 14 (14%) scored in the 11–14 range indicating moderate depression, and 2 (2%) scored above 15 indicating severe depression (Stern, 2014).

Mean scores out of 0–100 possible for the four language summary measures were analytic, 31.54 (*SD*: 11.01); authentic, 12.92 (*SD*: 10.58); clout, 85.64 (*SD*: 9.22); and emotional tone, 91.12 (*SD*: 11.53). Correlations between the language summary measures and adjusted HADS depression scores were analytic, *r* = 0.13, *p* = 0.203; authentic, *r* = − 0.02, *p* = 0.813; clout, *r* = − 0.24, *p* = 0.014; emotional tone, *r* = − 0.35, *p* < 0.001 (Table 3). The two measures with statistically significant correlations with HADS depression, clout and emotional tone, were considered as potential observed indicators of a language behavior latent construct, as these two language indicators were relevant to depression. The distribution of emotional tone scores was highly skewed, with a median = 98.16 indicating over half of the scores were greater than 98% and thus emotional tone was not included in the SEM analyses. The distribution of clout scores had a median of 87.1% with a non-significant Shapiro–Wilk (*p* = 0.560) indicating that the underlying sampling distribution was normally distributed, and clout was included in the SEM analyses.

The observed indicators of the latent construct, compassion capacity, were compassion satisfaction, burnout, and empathy. Burnout was negatively correlated with compassion satisfaction, *r* = − 0.63, *p* < 0.001; and empathy, *r* = − 0.23, *p* = 0.021. The correlation between compassion satisfaction and empathy did not reach statistical significance, but the relationship was in the predicted direction, *r* = 0.17, *p* = 0.088.

**Fig. 1:**
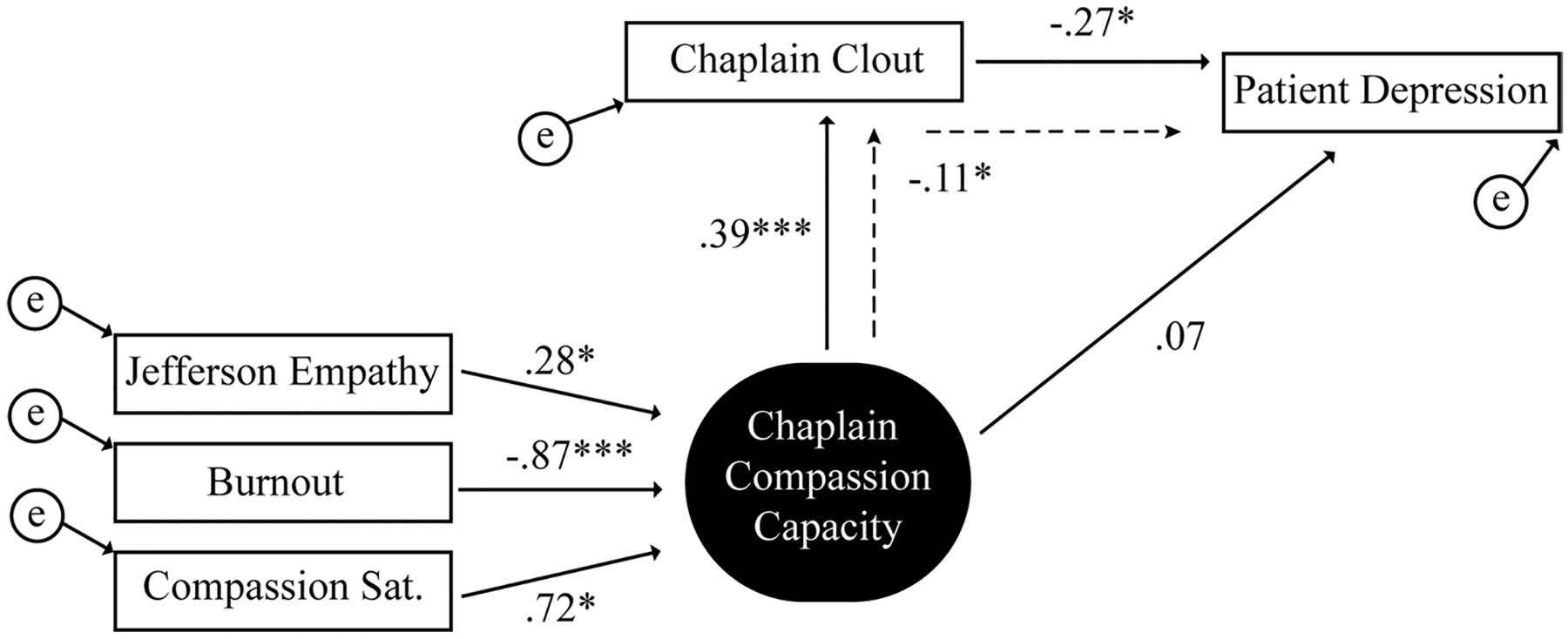
SEM model with chaplain compassion capacity, chaplain language, and patient depression (controlling for pre-consultation distress). The model revealed a standardized effect of chaplain compassion capacity on language (*β* = .39, *p* < .001), that chaplain language predicted patient depression (*β* = − .27, *p* = .013), and that compassion capacity exerted an indirect effect (dashed line) on patient depression through chaplain clout (*β* = − .11, *p* = .017). *p ≤ 0.05 and ***p ≤ 0.001. *P* values are two-tailed. Solid arrows indicate direct effects. Dashed arrows indicate indirect effects

In Fig. 1, the SEM model displays the mechanism of chaplain compassion capacity, chaplain language, and patient depression. With 11 distinct parameters estimated, the model indicated a good fit: *χ*^2^/df = 7.86, *p* = 0.097; RMSEA = 0.098 (90% CI, 0.000, 0.200); CFI = 0.948. The model revealed a standardized effect of chaplain compassion capacity on language (*β* = 0.39, SE = 0.28, *p* < 0.001) and that chaplain language predicted patient depression (*β* = − 0.27, SE = 0.01, *p* = 0.013). There was not a significant direct effect from compassion capacity to patient depression (*p* = 0.533) but using bootstrapping (bias-corrected method) to generate indirect effects, the model revealed a standardized indirect effect of compassion capacity on patient depression via chaplain language (*β* = − 0.11, SE = *p* = 0.017).

## Discussion

Based on a wealth of research demonstrating associations between physical health and psychosocial well-being, modern healthcare is increasingly characterized by a patient-centered model of care that places a premium on the compassionate treatment of the patient as a physical, psychosocial, and spiritual whole (Clark, 2007; Puchalski & Ferrell, 2011). However, the implementation of this patient-centered model of care is impeded by pressures endemic to most US healthcare systems, such as organizational shifts that limit the time for doctor-patient interactions (Gottschalk & Flocke, 2005; Weigl et al., 2009), the demands of electronic medical recording (Babbott et al., 2014; Block et al., 2013; Haider et al., 2018; Hill et al., 2013; Snowden & Kolb, 2017), and an epidemic of chronic disease (Østbye et al., 2005). Such pressures leave healthcare personnel overburdened (Dyrbye & Shanafelt, 2011) and all-too-often unable to adequately meet patients’ socio-emotional and spiritual needs (Lown et al., 2011; Tehranineshat et al., 2019). It is in this context that hospital chaplains play a critical role in reducing patient distress and bolstering patient resilience, positive emotions, and well-being, highlighting the importance of research to understand how chaplains deliver compassionate spiritual healthcare. Here, we found that a latent indicator of chaplains’ self-reported compassion capacity was significantly associated with their levels of clout, a measure of linguistic behavior reflecting expressive confidence, inclusivity, and other-centeredness. Chaplains’ clout scores, in turn, were associated with lower levels of patient depression after the consultation. Of note, chaplain-reported compassion capacity was not directly associated with patient benefit, but it exerted statistically significant indirect effects on patient depression via increased clout language (Hayes, 2009).

The fact that chaplains scoring high in the compassion capacity latent variable had higher clout scores and were of more benefit to patients warrants further thought as to what high clout scores may indicate about the chaplain. The clout measure within the LIWC analytic platform is based on a previous set of studies that identified language predictive of social status (Kacewicz et al., 2014). Specifically, this set of studies found that people of higher status tend to use different pronouns: fewer first-person singular (e.g., *I*, *me*) and impersonal pronouns (e.g., *it*); more first-person plural (e.g., *we*) and second-person singular pronouns (e.g., *you*). In addition, they use more social words (e.g., *everybody*, *family*) and fewer negation words (e.g., *never*, *shouldn’t*) (Kacewicz et al., 2014). It is important to note that the clout measure identified by Kacewicz and colleagues is not tapping into a *desire* or *grasping* for status, clout, or power; rather, it is derived from research interrogating linguistic behavior exhibited by people who demonstrate relatively high levels of leadership, confidence, and status in interpersonal interactions. The authors concluded that clout scores reflect the finding that “higher status individuals focus their attention outward, toward the person they are speaking with” and they state that, “those in a higher position in the hierarchy are more other-focused, whereas those lower in the social hierarchy are more self-focused as gauged by the use of personal pronouns” (Kacewicz et al., 2014). Extending these findings, another recent study identified negative associations between clout and LIWC categories reflecting discrepancy, certainty, and differentiation. It is possible that the combination of confidence and attunement to others expressed in clout language contributes to a sense of safety for patients, reflecting a provider who is competent as well as responsive and attentive (Moore et al., 2021).

It is clear from previous research that pronoun use is also highly associated with the mental, physical, and interpersonal health of the speaker. For example, one study found that people who use more first-person singular pronouns in trauma narratives constructed within 18 days of trauma had greater symptoms of post-traumatic stress disorder 6 months after the trauma, even when controlling for baseline symptom severity and verbal intelligence levels (Kleim et al., 2018). Among adults with a trauma history who are recently divorced, greater use of first-person singular pronouns predicted future psychological distress (Borelli & Sbarra, 2011). Another study found that people with high levels of interpersonal distress and an intrusive conversational style use more first-person singular pronouns (Zimmermann et al., 2013). A robust literature, including a meta-analysis of almost 5000 people from multiple countries, links I-talk with negative emotionality and depression (Tackman et al., 2019). Together, these findings indicate that less frequent use of first-person singular pronouns is associated with overall better psychological health and well-being. Our findings add to this by showing that chaplains with greater self-reported compassion capacity use language reflective of an other-oriented interpersonal style, including fewer first-person singular pronouns and more plural pronouns. By showing that compassion capacity is associated with relatively more other-oriented language, this finding also extends research linking positive mental health with compassionate person-centered care in a clinical environment (Cherry et al., 2013).

This study also adds to what is known about how patient outcomes are associated with the linguistic behavior of those who care for them, and our findings are consistent with an emerging body of research that links the use of plural pronouns (“we talk”) from family and caregivers with positive health outcomes among patients. For example, one influential study examined how patients and their romantic partners discussed their coping strategies after diagnosis of heart failure and found that *we* talk by the spouse, but not the patient, predicted more positive clinical and psychosocial health (Rohrbaugh et al., 2008). Another study found that *we* talk by a spouse predicted whether a patient remained abstinent from smoking 12 months after completing a smoking cessation intervention (Rohrbaugh et al., 2012). In a clinical setting, patients were more adherent when their physician used fewer first-person singular pronouns in a pre-surgical consultation (Falkenstein et al., 2016). This is a critical finding, especially considering other research showing that physicians’ explicit racial bias is associated with their use of first-person singular pronouns with Black patients (Hagiwara et al., 2017). Another study found that, among romantic couples in which one partner is being treated for breast cancer, the spouse’s *we* talk was associated with better dyadic adjustment for the pair (Karan et al., 2017). Here, chaplains’ clout scores, largely indicative of their relative use of “we talk” and less frequent use of “I talk,” were associated with lower patient depression scores after the consultation, even after controlling for pre-consultation distress levels.

Given that burnout was a significant component to the “compassion capacity” latent construct, these findings also contribute to our understanding of the relationship between burnout and clinical care. Over the last decade, extensive research has highlighted the concerning prevalence of burnout among healthcare providers, as well as its harmful effects on clinical care and safety (Dyrbye & Shanafelt, 2011; Halbesleben & Rathert, 2008; Salyers et al., 2017; Shanafelt et al., 2005, 2010; Windover et al., 2018). More recent research has uncovered some of the psychosocial and cognitive mechanisms that mediate the relationship between burnout and suboptimal clinical care. For example, one recent study found that healthcare providers’ scores on the depersonalization subscale of the Maslach Burnout Inventory were associated with inaccuracy in characterizing emotional facial expressions, with providers high on depersonalization more likely to misclassify negative emotions as positive (Colonnello et al., 2021). Other research has identified an association between burnout and attention and memory bias for emotionally negative information (Bianchi et al., 2020; Sokka et al., 2014). Here we identify specific linguistic behavior associated with the relationship between burnout and reduced effectiveness among chaplains. That is, chaplains with higher burnout scores had lower levels of compassion capacity and lower clout scores, and their consultations resulted in a relatively reduced benefit to patients in terms of depression compared to chaplains with higher scores in these measures. Examining whether this finding holds among other types of healthcare providers will be an important next step.

Our findings also add to a present debate over the optimal role of compassion in medicine (Gilewski, 2015). On the one hand, scholars have argued that emphasizing compassion in the clinical environment may impart harm on clinicians, since healthcare providers may feel pressured into “emotional labor” that risks increasing or causing burnout (Smajdor, 2013; Wang, 2016). Others, however, have argued that compassion is a sustainable alternative to and may even be antithetical to the empathic distress that arises when a clinician becomes overwhelmed by self-oriented negative emotions (Klimecki et al., 2013; Singer & Klimecki, 2014). Our findings support the latter model, as compassion capacity was associated with linguistic behavior that is linked with confident leadership and that reflects a lack of self-centeredness. Our findings are also broadly consistent with a prominent line of thinking about medical empathy, which notes the importance of using emotional attunement to the patient in order to cultivate trust and to foster disclosure and discussion of information about the patient’s state that can be used to help (Halpern, 2003). This line of thinking also highlights the inhibitory role of anxiety and negative emotions in clinical empathy and emphasizes the importance of other-oriented focus that may be protective against personal distress (Ekman & Krasner, 2017). Ultimately, by pointing to the linguistic behaviors associated with compassion capacity, we believe these findings inform what is a long history of confusion and debate about the nature of clinical compassion, as well as about how compassion relates to a family of potential responses to distress that includes empathy, sympathy, and empathic concern (Chaney, 2020; Halpern, 2003).

While we found that emotional tone was highly correlated with post-consultation depression levels, we were unable to test the association between compassion capacity and emotional tone with SEM due to the high skew of the emotional tone variable. It is interesting in and of itself that chaplains used such high levels of positive language during consultations, a finding that warrants future research. Moreover, chaplain emotional tone was not significantly correlated with pre-consultation distress, but it was inversely correlated with post-consultation depression levels. This raises the intriguing possibility that the extent to which chaplains use positive emotion language is predictive of patient benefit from the consultation.

Previous research has examined the use of affective language in the clinical environment, for example, finding that patients report liking their physician more if the provider used fewer negative emotion words (Falkenstein et al., 2016). Another study found that, in romantic couples in which one partner was recently diagnosed with breast cancer, the cancer patient had relatively better dyadic adjustment when their partner used more positive and fewer negative emotion words (Karan et al., 2017). Other studies have found that people who use relatively more positive emotion words compared with negative emotion words while writing about a past trauma had more positive health when tracked over time (Pennebaker et al., 1997). Together, this body of research indicates that the use of relatively more positive emotion words than negative emotion words is associated with better psychosocial outcomes. While we are unable to definitively test this association with our data, our findings are consistent with this body of research that finds an association between affective language and patient outcomes.

Here, we focused on only one patient outcome, namely depression, given the importance of depression for patient well-being and long-term clinical prognosis (Beeler et al., 2020; Froiland, 2008; Pinquart & Duberstein, 2010; Satin et al., 2009). Moreover, depression is highly prevalent among hospitalized patients due in part to factors often inexorable from the experience of serious illness such as medical uncertainty, anxiety, and pessimism (Caruso et al., 2017; Giammanco & Gitto, 2016; Kuhnt et al., 2016; McKenzie et al., 2010; Singer et al., 2009; Walker et al., 2018). In our current study, 23% of the patients met the criteria for some level of depression after the consultation, with 16% reporting moderate to severe levels. Almost 75% of patients met the criterion for clinically significant distress before the chaplain consultation, making it critical to understand how chaplains and other clinicians can address such highly pervasive suffering. However, depression is certainly only a single facet of the complex emotional experience of hospitalized patients, and future research approaches will be important for examining how compassionate language promotes psychological well-being and flourishing, even in the face of serious illness (Folkman & Greer, 2000).

### Limitations and Future Research

The findings from this study indicate that training in the skills, attributes, and virtues that result in increased clout language may be an effective means for increasing skillful and effective compassion within clinical healthcare; however, caution is warranted for several reasons. The sample size used here may have been underpowered to adequately evaluate other possible models. In addition, these data were collected from 16 chaplain residents providing spiritual consultations in one hospital system. While some of the chaplains were multi-lingual, we did not assess linguistic backgrounds and the study was limited to chaplain-patient interactions in English. Nor did we assess chaplains’ religious affiliations. It is not clear that these findings would be generalizable to spiritual consultations conducted in other regions or cultures. Related, we are not able to do a factor analysis of the compassion capacity construct with so few participants. However, the three measures are correlated with one another in a direction consistent with what would be expected, and all three have a significant relationship with the latent construct. Future larger studies will be important to examine and extend the associations identified in this study more robustly.

Moreover, while we believe these data reveal important new knowledge about skillful compassion in a clinical environment, caution is warranted against becoming too reductionist or prescriptive based on data such as these. First, we did not examine the context or themes associated with major linguistic categories, which makes definitive interpretation challenging. In fact, a recent study found that physicians’ levels of implicit racial bias were associated with the use of first-person plural words used with Black patients, a finding they interpreted as reflecting a more authoritative style (e.g., “We need to make sure that you take care of yourself.”) (Hagiwara et al., 2017). While we interpreted the current finding that the use of first-person plural words was associated with more positive patient outcomes and chaplain compassion as reflecting a prosocial and other-oriented style, from the current analysis we cannot determine how chaplains tended to use these pronouns. Future studies could interview patients to examine their experience of the chaplain consultation which, coupled with the methods used here, has the potential to connect chaplain compassion capacity and behavior with the phenomenology of receiving compassion. Of note, another limitation is that because patient language was not entered into the analyses reported here, we did not examine chaplain residents’ interactive responses to patients as has been done in previous studies (e.g., Levinson et al., 2000; Suchman et al., 1997). The therapeutic alliance between chaplain and patient is a co-created didactic relationship, and future research using LIWC can uncover the rich ways that language plays a role in establishing and maintaining this alliance. Moreover, here the focus was on linguistic behavior, which is by no means the only way that compassion is manifest in the clinical environment (Brugel et al., 2015; Halpern, 2014; Schattner, 2009; Singh et al., 2018). Chaplains’ use of eye contact, body language, therapeutic touch, facial expressions, and quiet presence are crucial conveyors of compassion that cannot be measured with the methods used here, but an assessment of these nonverbal behavioral markers of compassion could be combined with linguistic measures to get a more comprehensive understanding of chaplain compassion and its impact of patient outcomes.

Ultimately, these findings point to new metrics that may inform compassion and communication skills training in medicine. Perhaps the most important implication of our findings is that chaplain-reported compassion capacity, alone, was not enough to shift patient outcomes. Rather, compassion capacity benefited patients when it was “activated” by linguistic behavior, translating self-reported compassion into an effective clinical encounter. While a spate of recent research has been conducted to evaluate interventions that aim to cultivate clinical compassion (Banerjee et al., 2021; Sinclair et al., 2021, Sinclair et al., 2021a, 2021b, 2021c; Sinclair et al., 2021; Sinclair, Jaggi, et al., 2021), less research has examined how skills and attributes are manifest by behaviors that convey and impart compassion and very few studies have examined linguistic or patient outcomes related to training (Banerjee et al., 2021). Examining whether the relationship between compassion capacity, clout, and lower patient depression holds for other types of clinicians is an important next step in this line of research. Recent research highlights the importance of creating a culture of compassion that is infused throughout the healthcare system (Sinclair, et al., 2021a, 2021b, 2021c), and the study design used here could be leveraged to explore and compare the language of compassion across disease types, demographic groups, and clinician roles. Understanding how and why patients feel held in care is critical, especially for patient groups experiencing high levels of stigma or complex illness (Cathébras, 2021; Williamson et al., 2021).

## Figures and Tables

**Table 1:** Social and demographic characteristics of chaplain residents

		*N*	%
Gender			
	Female	10	63%
	Male	6	38%
Race			
	Asian	2	13%
	African American/Black	10	63%
	Afro-Caribbean	1	6%
	White	2	13%
	Other	1	6%
Relationship			
	Single	6	38%
	Divorced	3	19%
	Single, living with someone	7	44%

**Table 2:** Clinical, social, and demographic characteristics of patients

		*N*	%
Sex			
	Female	57	56%
	Male	44	44%
Race			
	Asian	3	3%
	Black	47	47%
	White	47	47%
	Unknown	4	4%
Relationship			
	Married	45	45%
	Divorced	12	12%
	Separated	1	1%
	Single	33	33%
	Widowed	8	8%
	Unknown	2	2%
Admitting Hospital Service			
	Unknown	15	15%
	General medicine	20	20%
	Cardio/cardiovascular	2	2%
	Emergency/ICU	34	34%
	Urology/gynecology	4	4%
	Neurology/neurosurgery	11	11%
	Psychiatry	4	4%
	Rehab medicine	5	5%
	Thoracic/pulmonary	6	6%
Religion			
	Unknown	9	9%
	Protestant Christian	72	72%
	Catholic	4	4%
	Muslim	1	1%
	Jewish	1	1%
	No preference	14	14%

**Table 3:** Pearson correlations between variables comprising the compassion capacity latent variable (compassion satisfaction, burnout, Jefferson empathy), linguistic summary variables, patient distress prior to the consultation, and post-consultation depression residuals (controlling for pre-consultation distress)

	Compassion sat	Burnout	Jefferson emp	Clout	Emotional tone	Pre-con. distress	Depression
Compassion sat		− .633***	.171	.264**	.127	− .040	− .063
Burnout	− .633***		− .229*	− .331***	− .096	.135	.009
Jefferson emp	.171	− .229*		.332***	.021	− .011	− .014
Clout	.264**	− .331***	.332***		.183	− .011	− .238*
Emotional tone	.127	− .096	.021	.183		− .194	− .345***
Pre-con. distress	− .040	.135	− .011	− .011	− .194		.000
Depression	− .063	.009	− .014	− .238*	− .345***	.000	

*** Correlation is significant at the .001 level (2-tailed)

** Correlation is significant at the 0.01 level (2-tailed)

* Correlation is significant at the 0.05 level (2-tailed)

## Data Availability

All data analyzed for this study are available at the Open Science Framework (https://osf.io/cgq8z/).
